# Gastric ulcer induced changes in substance P and Nk1, Nk2, Nk3 receptors expression in different stomach localizations with regard to intrinsic neuronal system

**DOI:** 10.1007/s00418-018-1715-4

**Published:** 2018-08-28

**Authors:** Michal Zalecki

**Affiliations:** Department of Animal Anatomy, Faculty of Veterinary Medicine, University of Warmia and Mazury in Olsztyn, Oczapowskiego 13 str., 10-719 Olsztyn, Poland

**Keywords:** Substance P, Tachykinin receptors, Stomach ulcer, Enteric nervous system

## Abstract

Gastric ulceration, a focal tissue damage accompanied by inflammation, can influence other parts of the stomach. Substance P and its receptors are strongly involved in regulation of gastrointestinal motility, secretion and inflammation. The enteric nervous system is one of the regulators of gastrointestinal functioning and contributes to tissue response to the pathology. The pig, an omnivorous animal, is a valuable species for gastrointestinal experiments. Thus, the objective of the study was to verify whether the antral ulceration induces changes in the expression of substance P and tachykinin receptors in the neighboring (antrum) and distanced (corpus, pylorus) porcine gastric tissues and therein localized myenteric and submucosal perikarya as well as in the intrinsic descending neurons supplying pyloric sphincter. The experiment was performed on healthy pigs and pigs with experimentally induced gastric ulcers. Stomach samples from the corpus, antrum (adjacent to the ulcer in experimental pigs) and pylorus were analyzed by: (1) double immunofluorescence for changes in the number of SP-positive myenteric and submucosal neurons (2) Real-Time PCR for changes in expression of mRNA encoding SP and Nk1, Nk2, Nk3 receptors. Additionally, gastric descending neurons supplying pyloric sphincter were immunostained for SP. In experimental animals, only the number of SP-positive myenteric perikarya significantly increased in all stomach localizations studied. Q-PCR revealed increased expression for: SP, Nk1, Nk3 in the corpus; Nk2 and Nk3 in the pylorus; In the antrum, expression of Nk3 was increased but Nk2-decreased. Antral ulcers induced significant changes in the expression of SP and tachykinin receptors in the wide stomach area indicating sophisticated tissue reaction.

## Introduction

Gastric ulceration is a disorder commonly occurring in humans and in other mammals including pigs (Najm 2011). Although gastric ulcerations are known for many centuries (Baron 1998), most of “gastrointestinal tachykinin subjected” basic science studies were focused on the exploration of intestinal pathologies (Mantyh et al. 1994, 1995, 1996, 1988; Kaleczyc et al. 2010; Pidsudko et al. 2008; Sienkiewicz et al. 2006). Publications dealing with the involvement of neuronal elements and biologically active substances in gastric ulcer disease are very sparse. Swine as an omnivorous animal exhibiting many similarities to human organism, especially as concerns the gastrointestinal tract, represents a highly valuable animal model for biomedical research (Swindle 2012). Although factors contributing to the development of gastrointestinal ulcers are multiple, the pathological changes at a certain stage of the disease are similar (Najm 2011). The ulcer cavity is surrounded by acute and/or chronic inflammation, however, the ulcer itself, as a focal pathological process, differs from other gastrointestinal inflammatory processes that usually affect large parts of the mucosa. Nevertheless, the gastrointestinal tract is characterised by the unique reaction mechanism in which the tissues widely distanced from the direct injury could also respond (Lomax et al. 2005). Thus, the ulcer localized in e.g. the gastric antrum can additionally elicit a response of tissues (including intramural neurons and intrinsic neural pathways) found in other, proximal and distal, regions of the stomach (not directly covered by the damage).

The enteric nervous system is a main and direct regulator of the motility and secretion processes occurring in the gastrointestinal tract (Furness 2006; Surprenant 1994; Timmermans et al. 1992). It represents a sophisticated system mesh which include descending and ascending intramural pathways (Furness et al. 1989). Intramural neuronal cell bodies controlling gastrointestinal motility are mainly localized in the myenteric plexus, while submucosal neurons are responsible for the mucosal secretion (Furness 2006; Timmermans et al. 1997). The morpho-functional arrangement of the stomach intramural nervous system was precisely described in different animal species (Reiche et al. 1998; Schemann et al. 2001; Schemann 2005) including the pig (Zacharko-Siembida and Arciszewski 2014; Van et al. 1996; Porcher et al. 2000; Kaleczyc et al. 2007; Rekawek et al. 2015; Bulc et al. 2015). Intramural neurons, due to their localization, are highly exposed to pathological processes affecting the gastrointestinal tract. Advancely developed gastric ulcers, which deeply penetrate into the muscular layers, directly influence the submucosal, myenteric and intramural nerve pathways.

Substance P and its receptors (Nk1, Nk2, Nk3) are widely accepted to be involved in inflammatory processes affecting different tissues and organs (O’Connor et al. 2004). According to many studies, SP is one of the key factors of the neuro-immune cross talk during inflammation (Vilisaar and Arsenescu 2016; O’Connor et al. 2004). The peptide is also extensively expressed in the enteric neurons during physiological conditions and it is known to regulate smooth muscles activity (Schmidt and Holst 2000).

In the view of such diverse functions of SP in the gastrointestinal tract, it can be assumed that changes in SP expression induced by inflammatory or other pathological processes can influence other gastrointestinal functions, such as the smooth muscles reactivity.

Therefore, the present study was aimed to verify the existence of an association between experimentally induced stomach antrum ulcers and the expression of substance P in gastric intramural neuronal perikarya (submucosal and myenteric), and levels of mRNA encoding Tac1 (substance P), and Nk1, Nk2 and Nk3 receptors in selected stomach localizations. The localizations covered the tissues directly adjacent to the ulcer (the stomach antrum) and those distanced from the inflammatory focus but found more proximally (the stomach corpus below the cardia) or distally (the pylorus at the level of the pyloric orifice). The expression of SP in intramural descending gastric neurons supplying the pyloric sphincter (the perikarya previously labelled with retrograde fluorescent tracer Fast Blue) was also investigated.

## Materials and methods

The handling of animals and all experimental protocols were submitted to and approved by the Local Ethics Committee of the University of Warmia and Mazury in Olsztyn (permit number 76/2012) affiliated to the National Ethics Commission for animal experimentation (Polish Ministry of Science and Higher Education). All the experimental procedures were performed under a license and in accordance with recommendations of the Ethics Committee.

The experiment was performed on sexually immature female pigs of the Polish Large White breed (body weight approx. 20 kg) obtained from a commercial fattening farm (14-260 Lubawa, Poland).

The pigs (*n* = 24) were randomly divided into the control (C, *n* = 12) and experimental (E, *n* = 12) groups. In the experimental animals, bilateral peptic ulcers were evoked in the stomach antrum by injections of 1 cm^3^ of 40% acetic acid solution into the submucosal layer of the stomach wall, according to the procedure by Okabe and Amagase (2005). All experimental procedures have been described in detail previously (Zalecki 2015).

Shortly, in the anesthetized pigs, stomachs were exposed via midline laparotomy and bilateral injections of acetic acid solution (1 cm^3^, 40%) were performed into the submucosal layer of the anterior and posterior wall of the antrum, about 1.5 cm from the pyloric orifice. To avoid leakage of the solution, a sterile tampon was tightly placed on the inserted needle at the time of the injection and left in the site for about 30 s after the needle removal. A weal-like swelling in the place of the injection confirmed the accuracy of the injection. Afterwards, the abdomen incision wound was sutured and the animals were moved to individual pens and kept under standard conditions for a period of 6 days. On the 7th day, the pigs were subjected to the final phase of the experiment and sacrificed.

Since the application of two different types of research techniques (double indirect immunofluorescence and Real-Time PCR) requires completely different fixation methods, after inducing antral ulceration the pigs were furtherly divided into two subgroups: (a) immunofluorescence group (IF-group) (*n* = 12; consisting of the control: *n* = 6 and experimental: *n* = 6, pigs)—the animals which were deeply anesthetized [as described previously (Zalecki 2015)], sacrificed and transcardially perfused with 4% paraformaldehyde in 0.1M phosphate buffer (pH 7.4). The tissues collected from this group were stained by immunofluorescence; (b) another group (*n* = 12; consisting of the control: *n* = 6 and experimental: *n* = 6) of pigs were deeply anaesthetized [as described previously (Zalecki 2015)] and exsanguinated—collected samples were used for molecular analysis (Real-Time PCR) (molecular group: M-group).

**Fig. 1:**
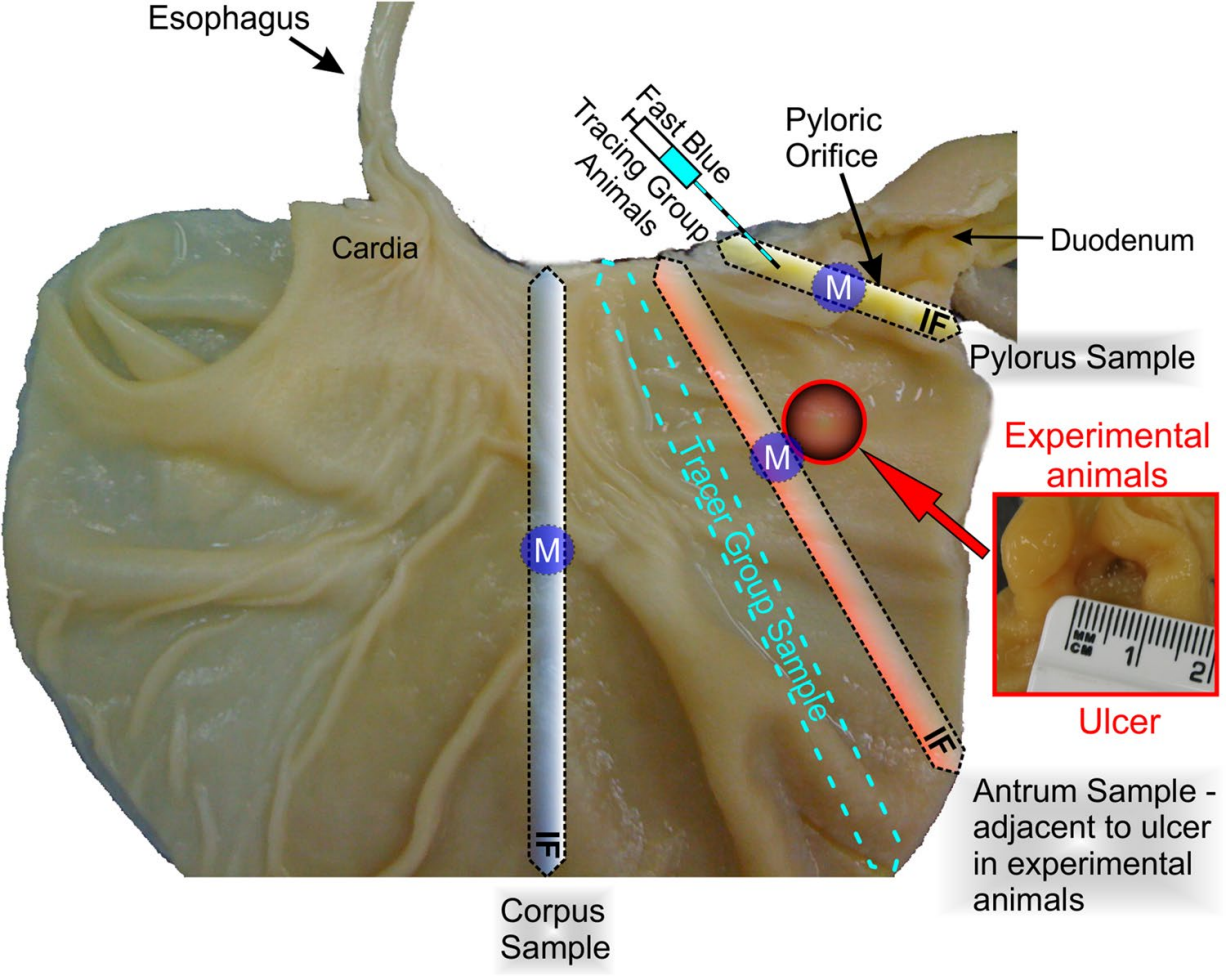
Drawing illustrating the method of tissue sampling in the immunofluorescence (IF), molecular (M) and tracing (T) animal groups. The markings are applied in the picture presenting the internal part of the stomach wall (view on the mucosa of the anterior wall). Ulcer localization (present in experimental animals) is indicated by the red circle. Picture showing the stomach ulcer (and its dimensions) is shown in the red frame. Samples of the stomach corpus (dotted line rectangle filled with grey gradient—with letters IF), stomach antrum (in experimental animals sections bordering with the ulcer) (dotted line rectangle filled with red gradient—with letters IF) and pylorus (dotted line rectangle filled with yellow gradient—with letters IF) were collected from the immunofluorescence group of animals (IF). Tissues for Real-Time PCR (violet circles with letter M) were cut out from the stomach corpus, stomach antrum (in experimental animals sample slices bordering with the ulcer) and pylorus from the animals of the molecular group (M). Tissues containing Fast Blue (FB) traced perikarya were collected from the gastric antrum (blue dotted frame) of the tracing group pigs (T). Symbol of the syringe points to the Fast Blue tracer injection site (pyloric sphincter) in the T-group of animals

Stomachs collected from all the pigs were cut along the greater curvature and thoroughly washed in PBS to remove food debris. In the IF-group animals, 1 cm thick transverse section samples were taken from the following regions: the pylorus at the level of the pyloric orifice (Fig. 1, yellow frame); the wall adjacent to the ulcer—a part of the stomach antrum (Fig. 1, red frame); the stomach corpus below the cardia (Fig. 1, grey frame). The tissues were post-fixed in the same fixative as used for the perfusion (60 min.), rinsed in PBS for 2 days and transferred to and stored in 18% buffered (pH 7.4) sucrose solution for 3 weeks. 20 µm thick transverse cryostat consecutive tissue sections were cut and mounted on chrome alum–gelatine-coated slides, air-dried and stored desiccated at − 23 °C until further processing.

In the M-group pigs, two (bilateral) circular-shaped samples, having a diameter of 1 cm, were cut-out (by use of a round cutter) through the entire thickness of the stomach wall at sites strictly corresponding to those sampled in the IF-group animals (Fig. 1, blue circles with M): the pylorus; the region adjacent to the ulcer (antrum); and corpus. To ensure the correspondence of the tissue samples collected from each animal, the distances from the pyloric orifice were precisely measured in each animal studied and compared to those employed in the IF-group animals. Afterwards, the tissues were immersed in 4 °C RNAlater^®^ (Ambion, USA) overnight and finally stored at − 80 °C until processing.

Since the present experiment is a part of the wider study, the tissues containing gastric descending neurons supplying the pyloric sphincter and retrogradely traced with Fast Blue (FB) were collected during investigations precisely described in the previous article (Zalecki 2015). The pressure tracer injection technique, which enables precise application of Fast Blue with minimal damage to tissues (Oztas 2003), was applied in the study. Shortly: in the anaesthetized pigs, 20 µl of 5% aqueous suspension of fluorescent tracer Fast Blue (Polysciences, Inc; Cat# 17740) was injected with a Hamilton microsyringe into 4 places (about 5 µl into every place) equally distributed around the circumference of the pyloric sphincter wall. Following the needle insertion, the tracer was deposited throughout the entire thickness of the muscular layer, sliding the needle back towards the surface while gently and constantly pressing the syringe plunger. To avoid leakage of the tracer outside, the needle was left for about 30 s in the place of the injection. Finally, the surface of the pylorus was rinsed with physiological salt solution and gently drained with a sterile tampon. These procedures prevented non-specific labelling of the neurons. On the 7th day, the animals were again deeply anaesthetized, transcardially perfused with a 4% solution of paraformaldehyde in 0.1M phosphate buffer (pH 7.4) and the stomachs were collected from the control (*n* = 6) and experimental (*n* = 5) pigs. All further steps of sample preparation (post-fixation, rinsing, tissue sucrose infiltration, cryo-sectioning) were performed in accordance to the previously described protocol. Finally, the tissue samples were defined as the “tracing group” (T-group, *n* = 11, Fig. 1—blue dotted frame).

### Double indirect immunofluorescence

The immunofluorescence stainings were performed on tissues collected from the control and experimental pigs of group IF and T. The tissue sections obtained from the same stomach region in the particular animal were separated by a distance of at least 80 µm (greater than dimensions of the largest intramural perikarya) what provided security that none of the immunostained perikaryon was counted twice. The tissue slides were processed for routine double immunofluorescence stainings with mixtures of primary antibodies against pan-neuronal marker PGP 9.5 (mouse anti-PGP 9.5, dilution 1:600, code 7863-2004, clone 31A3, AbD Serotec) and substance P (rat anti-substance P, dilution 1:400, code 8450-0505, AbD Serotec), and corresponding secondary antibodies (AlexaFluor 488, goat anti-mouse, dilution 1:500, code A11001 and AlexaFluor 555, goat anti-rat, cross adsorbed, dilution 1:500, code A-21434, Invitrogen, USA). The primary antibodies used in the study were recommended and validated by the specific suppliers for application in the porcine tissues. All staining procedures and controls applied in the experiment were precisely described in the previous article (Zalecki 2012). Due to the lack of availability of primary antibodies designed for porcine tachykinin receptors and negative results obtained in own tests on some antibodies designed to such receptors of other species, the changes in receptors’ expression were determined exclusively by Real-Time PCR technique.

Microscopic analyses of immunolabeled sections were performed under the confocal microscope (LSM700, Zeiss), equipped with filter sets for AlexaFluor488, AlexaFluor555 and Fast Blue. During the analysis the investigator was blinded to experimental group—tissue slides were identified by laboratory technician and announced to investigator only after finishing analysis. To determine the percentages of SP-immunoreactive cells, the number of neurons simultaneously co-expressing pan-neuronal marker (or fluorescent tracer in case of T-group animals) and SP were counted. For tissues of the IF-group animals at least 400 of PGP 9.5-positive cell bodies in the myenteric and submucosal plexuses were analysed in each studied region of each stomach (counted in 5 microscopic tissue slides), what gave the sum of at least 2400 perikarya analysed in the particular animal. Although in the porcine stomach wall even two clearly distinguishable ganglionated submucosal plexuses with numerous neurons were described (Kaleczyc et al. 2007), there is a considerable variability in the occurrence and organization of these ganglia in different stomach locations. Therefore, all the neurons observed beneath the muscular layer were counted together and designated as the “submucosal plexus” perikarya.

In the T-group animals, at least 150 FB-positive neuronal somata from each stomach were analysed. The results were presented as average percentages ± SEM.

Photo-documentation has been prepared using a confocal laser microscope (LSM700, Zeiss) and its software (Zen 2009, ver. 5.5.0.282, Zeiss). Figures were composed into panels using Corel Draw X7 software (ver. 17.6.0.1021).

### Real-time PCR

The Real-Time PCR was applied on samples (pylorus, the antrum wall adjacent to the ulcer, corpus) collected from the control and experimental pigs in group M. To ensure the isolation of total RNA from all the stomach wall layers in each sample, the perpendicular section (containing all the layers) weighting 300 µg (taken from each circular-shaped tissue sample) was homogenized with 600 µl of fenozolone. Then, the appropriate volume of the liquid homogenate containing 50 µg of the tissue sample was used to isolate total RNA (Total RNA Mini Plus kit, A&A Biotechnology, Poland). This has provided certainty that all the samples in all the animals studied were unified regardless of the large thickness of the stomach wall in pig.

cDNA was generated using 1.5 µg of total RNA and Maxima First Strand cDNA Synthesis Kit for RT-qPCR (code K1672, Thermo Fisher Scientific) according to the manufacturer’s instruction. The mRNA expression levels for the following genes: Tac1 (encoding SP), Nk1, Nk2, Nk3 and porcine glyceraldehyde 3-phosphate dehydrogenase (GAPDH) as housekeeping gene were determined in doublets (for each cDNA sample) using 7500 fast Real-Time PCR system (Applied Biosystems, USA). Initial validation performed on samples obtained from experimental and control animals revealed that GAPDH was an appropriate and reliable housekeeping gene for the study (with stably expressed values for all the animals). The primers (Table 1) were designed using sequences of origin available in Gen Bank and Primer-BLAST software (http://ncbi.nlm.nih.gov). The protocol of the reaction was as follows: 10 min initial denaturation on 95 °C, 15 s denaturation on 95 °C, and 1 min annealing on 60 °C for 40 cycles. The data for Tac1 (SP), Nk1, Nk2, Nk3 expression were normalised against GAPDH using software 7500 v. 2.0.2 (Applied Biosystems, USA).

**Table 1:** Sequences of primers used in the Real-Time PCR

Gene	Sequences of primers	Sequence of origin (in Gene Bank)
GAPDH	Forward: TTCCACCCACGGCAAGTT Reverse: GGCCTTTCCATTGATGACAAG	XR_002343817.1
Tac1 (SP)	Forward: GCGACCAGATAAAGGAGGAGCTGC Reverse: CTGCTGAGGCTTGGGTCTCCG	XM_003130164.6
Nk1	Forward: GACTTCCGTGGTGGGCAACG Reverse: GGCCATGGAGGCCTCCGCGA	XM_021087317.1
Nk2	Forward: GCCTCACGCTCTGGAGACGC Reverse GGCAGCCAGCAGATGGCGAA	XM_001929073.7
Nk3	Forward: ACCCGGGGCTGCAGACCTAC Reverse: AGGCTGGAGCGCTACGCTCA	XM_003482466.3

Primers were designed using sequences of origin available in Gen Bank and Primer-BLAST software (http://ncbi.nlm.nih.gov)

### Statistical analysis

The differences in the number of SP-immunoreactive cells and expressions of mRNA encoding studied genes between the control and experimental animals were statistically analyzed with GraphPad Software Inc., USA, ver. 6. First, the values were tested with D’Agostino and Pearson omnibus normality test to verify if they come from a Gaussian distribution. Then, the values were analyzed using Student’s *t* test (or Mann–Whitney *U* test for non-normal distributed data), and considered to be significant at *P* < 0.05. Finally, using G*Power 3.0.10 software (Franz Faul, Universitat Kiel, Germany) the power of statistical tests was verified (for selected results indicating statistically significant differences).

## Results

**Fig. 2:**
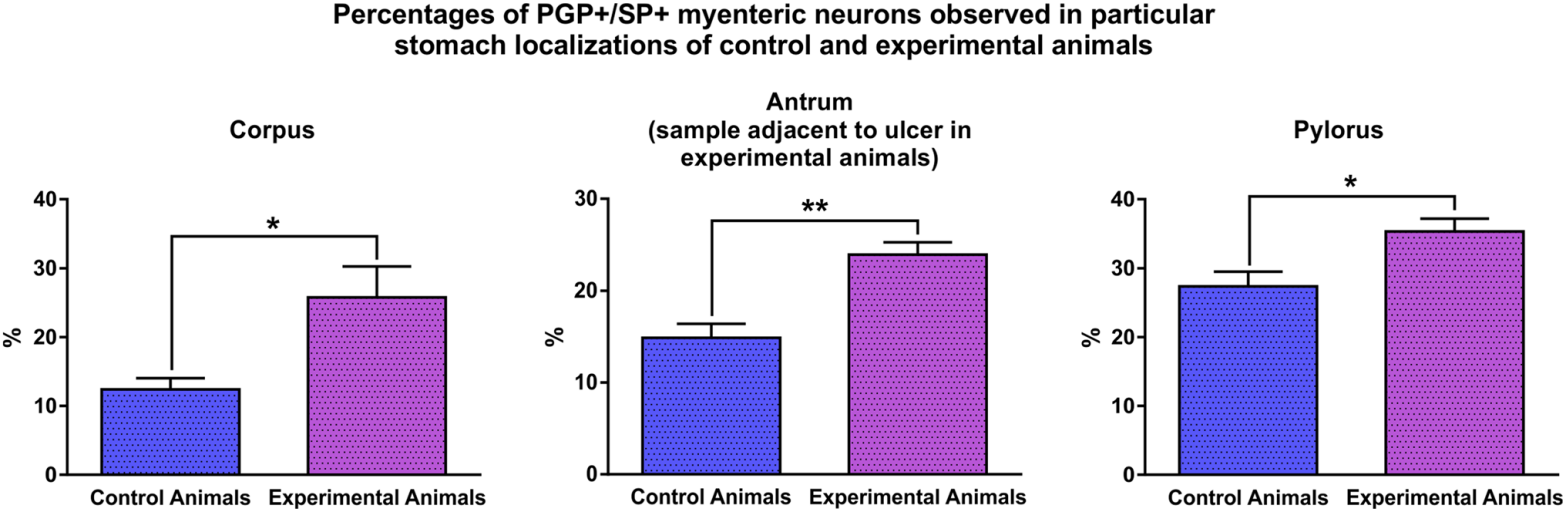
Graph showing the percentages of PGP+/SP + myenteric perikarya observed in the stomach corpus, stomach antrum (in experimental animals sections adjacent to the ulcer) and pylorus in the control and experimental animals of the IF-group. Statistically significant differences existing between the control and experimental animals (in particular stomach localizations) are marked by asterisks, *P ≤ 0.05, **P ≤ 0.01

**Fig. 3:**
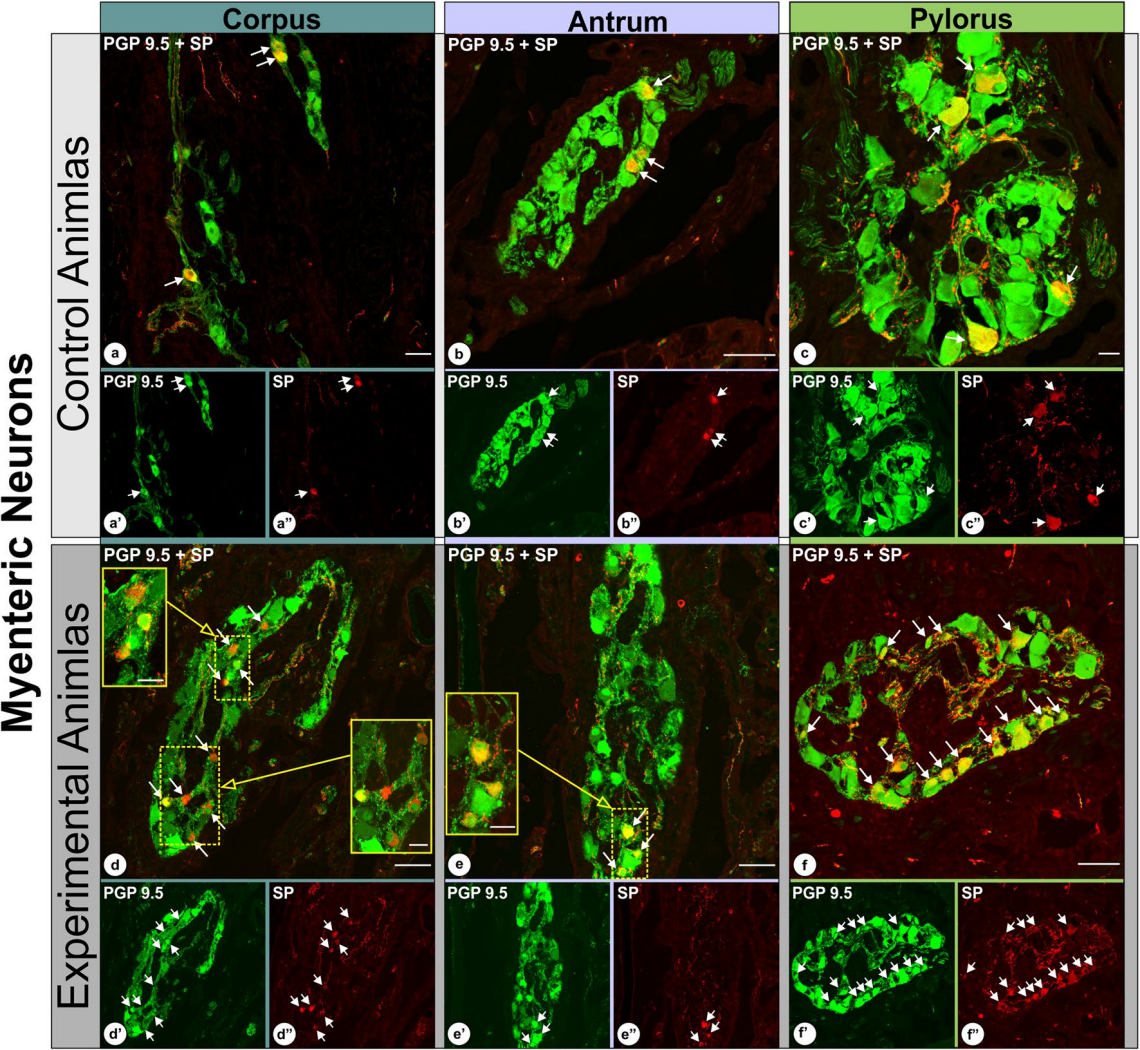
Set of microphotographs showing PGP 9.5/SP-immunoreactive myenteric perikarya in the control (**a, a**′, **a**″; **b, b**′, **b**″; **c, c**′, **c**″) and experimental (**d, d**′, **d**″, **e, e**′, **e**″; **f, f**′, **f**″) animals in the following stomach cross-sections: corpus (**a, a**′, **a**″; **d, d**′, **d**″), antrum - at the level bordering with the ulcer in experimental animals (**b, b**′, **b**″; **e, e**′, **e**″) and pylorus (**c, c**′, **c**″, **f, f**′, **f**″). Presented representative microscopic cross-sections were taken from IF-group animals and double-immunolabelled with antibodies against PGP 9.5 (**a**′, **b**′, **c**′; **d**′, **e**′, **f**′) and substance P (**a**″, **b**″, **c**″; **d**″, **e**″, **f**″). Pictures (**a, b, c; d, e, f**) present overlap of both fluorescence channels. Arrows point to perikarya that simultaneously co-expressed PGP 9.5 and SP immunoreactivity. High magnification pictures of the selected areas [dotted line frames in the pictures (**d**) and (**e**); scale bar size: 20 µm] precisely show selected neurons. Scale bar size: 50 µm (**a, b, d, e, f**); 20 µm (**c**)

**Fig. 4:**
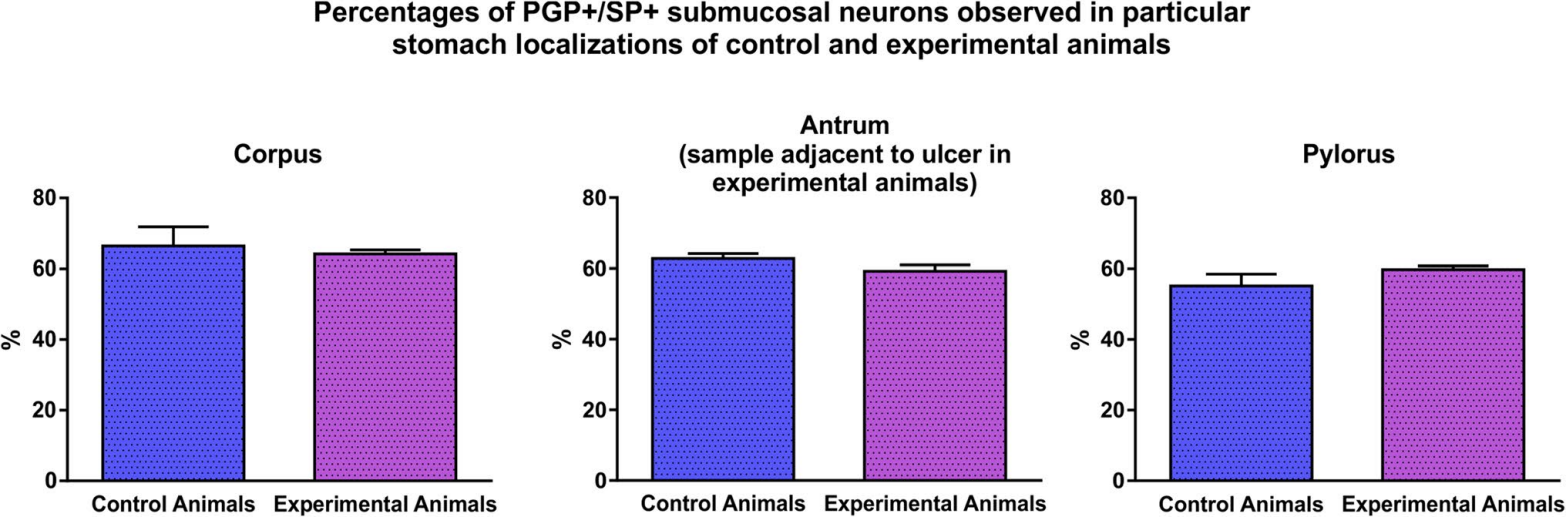
Graph showing the percentages of PGP+/SP + submucosal perikarya observed in the stomach corpus, stomach antrum (in experimental animals sections adjacent to the ulcer) and pylorus in the control and experimental animals of the IF-group. There were no statistically significant differences between the control and experimental animals observed

**Fig. 5:**
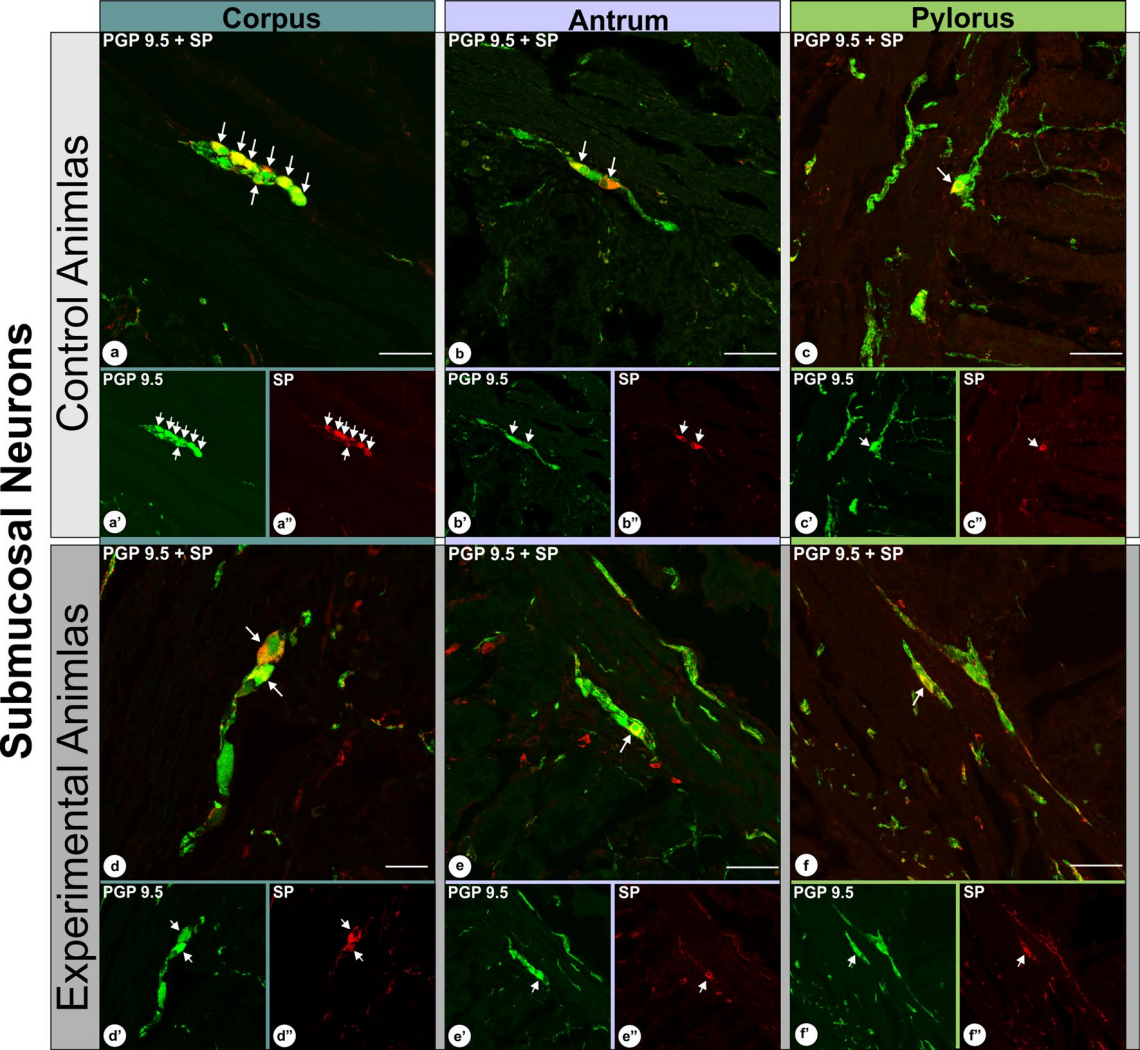
Set of microphotographs showing PGP 9.5/SP-immunoreactive submucosal perikarya in the control (**a, a**′, **a**″; **b, b**′, **b**″; **c, c**′, **c**″) and experimental (**d, d**′, **d**″, **e, e**′, **e**″; **f, f**′, **f**″) animals in the following stomach cross-sections: corpus (**a, a**′, **a**″; **d, d**′, **d**″), antrum—at the level bordering with the ulcer in experimental animals (**b, b**′, **b**″; **e, e**′, **e**″) and pylorus (**c, c**′, **c**″, **f, f**′, **f**″). Presented representative microscopic cross-sections were taken from IF-group animals and double-immunolabelled with antibodies against PGP 9.5 (**a**′, **b**′, **c**′; **d**′, **e**′, **f**′) and substance P (**a**″, **b**″, **c**″; **d**″, **e**″, **f**″). Pictures (**a, b, c; d, e, f**) present overlap of both fluorescence channels. Arrows point to perikarya that simultaneously co-expressed PGP 9.5 and SP immunofluorescence. Scale bar size: 50 µm (**a, b, c, e, f**); 20 µm (**d**)

The analysis of double-immunolabeled sections obtained from IF-group pigs revealed that in the experimental animals the number of SP-positive neurons significantly increased in the myenteric plexus in all the regions studied, while the fluctuations in the number of SP-immunoreactive submucosal neurons, although occurred, were not statistically significant. In detail: the percentage of SP-immunoreactive myenteric neurons observed in the control animals (Fig. 2) amounted to 12.4 ± 1.6% in the stomach corpus (Fig. 3a, a′, a″), 14.9 ± 1.5% in the stomach antrum [at the level corresponding to that found in the ulcer boundary in the experimental animals] (Fig. 3b, b′, b″) and 27.4 ± 2.1% in the pylorus (Fig. 3c, c′, c″). In the experimental animals (Fig. 2) these numbers were 25.8 ± 4.5% (Fig. 3d, d′, d″), 23.9 ± 1.3% (Fig. 3e, e′, e″) and 35.3 ± 1.9% (Fig. 3f, f′, f″), respectively, and all these changes were statistically significant (Fig. 2). The number of SP-immunopositive submucosal neurons in the control animals (Fig. 4) amounted to 67.1 ± 5.4% in the stomach corpus (Fig. 5a, a′, a″), 62.8 ± 1.4% in the stomach antrum [at the level corresponding to that found in the ulcer boundary in the experimental animals] (Fig. 5b, b′, b″) and 55.2 ± 3.3% in the pylorus (Fig. 5c, c′, c″), while in experimental animals (Fig. 4) these values were 64.2 ± 1.1% (Fig. 5d, d′, d″), 59.3 ± 1.7% (Fig. 5e, e′, e″) and 59.8 ± 1.0% (Fig. 5f, f′, f″), respectively, and the fluctuations were not statistically significant (Fig. 4).

**Fig. 6:**
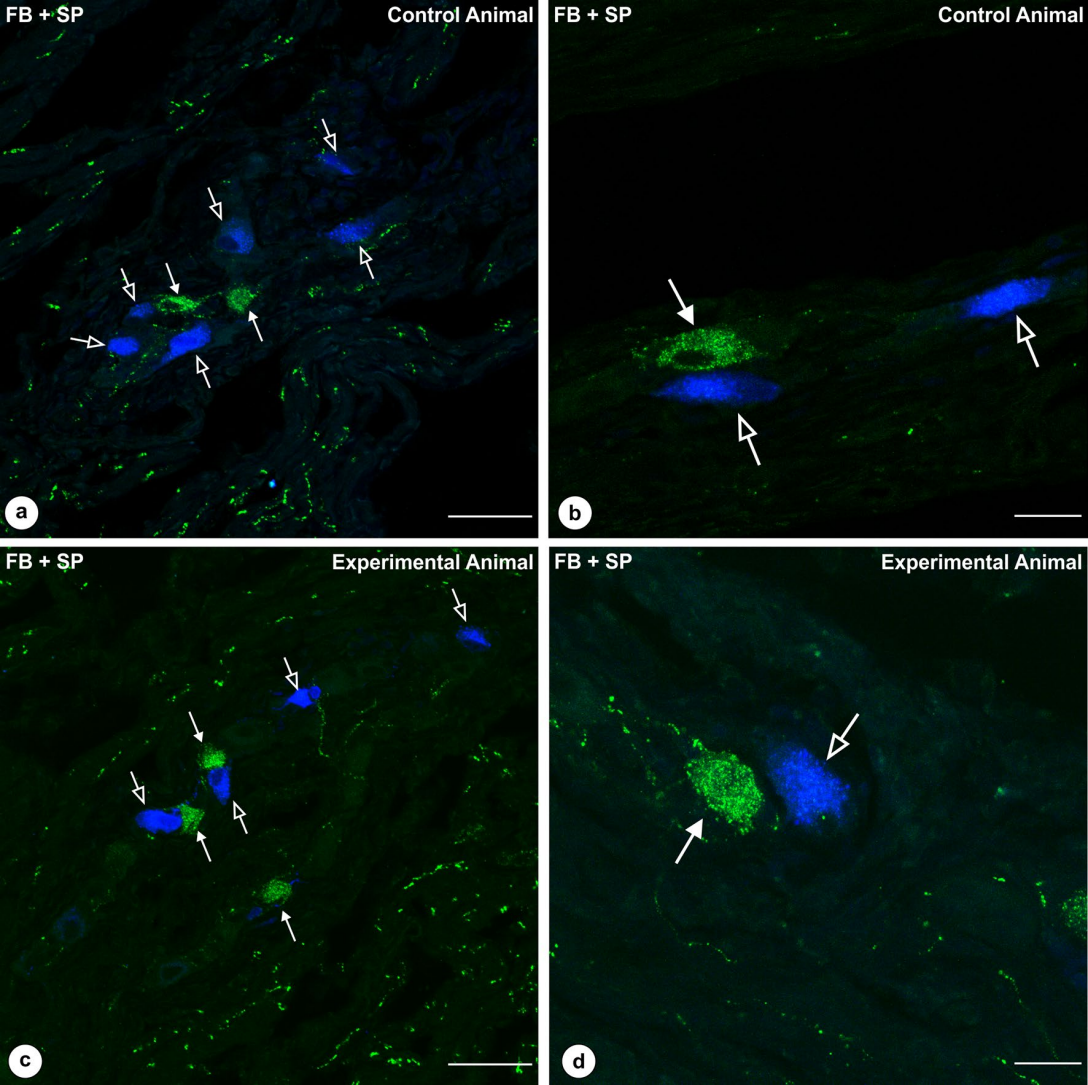
Set of microphotographs showing stomach antrum sections with FB-positive myenteric neurons collected from animals of the group T and immunostained with antibodies against substance P. None of FB-positive perikarya (empty arrows, blue) in the control (**a, b**) and experimental (**c, d**) animals expressed immunoreactivity to substance P (solid arrows, green), while some of the traced neuronal somata were adjacent to SP-immunoreactive cells (**b, d**). Scale bar size: 50 µm (**a, c**); 20 µm (**b, d**)

The detailed analysis of the antral sections obtained from T-group animals revealed that none of the retrogradely traced neurons expressed SP-immunoreactivity in the control and experimental pigs. However, in a close proximity to some of the traced neurons, SP-immunoreactive perikarya or nerve fibers were observed (Fig. 6a–d).

**Fig. 7:**
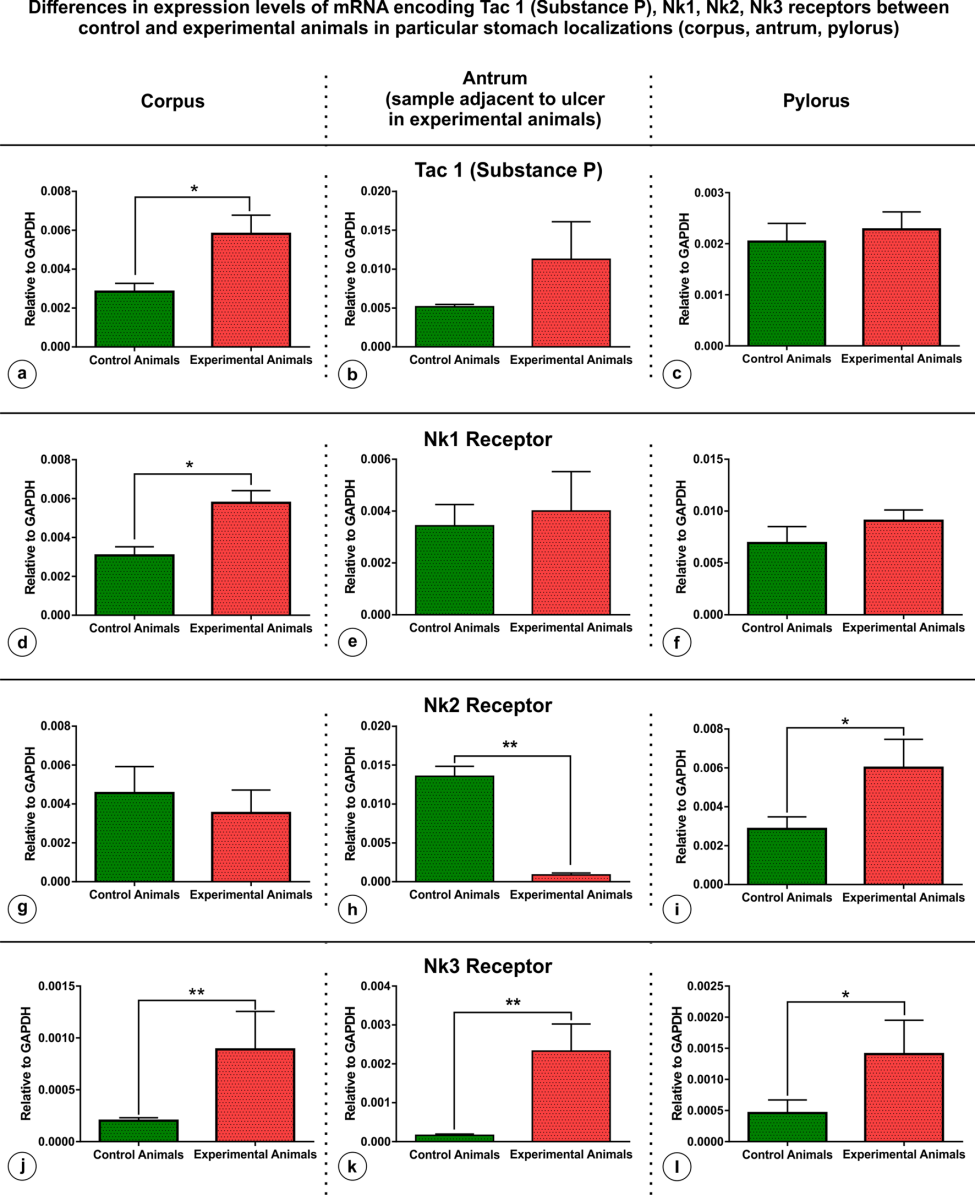
Graph showing the differences in expression levels of mRNA encoding Tac1 (Substance P), Nk1, Nk2 and Nk3 receptors in the stomach corpus, stomach antrum (in experimental animals sections adjacent to the ulcer) and pylorus existing between control and experimental animals of the group M. Levels of Tac1, Nk1, Nk2, Nk3 mRNA were measured by Real-Time PCR. The data obtained from each sample were normalized to GAPDH. Relative quantities (RQ) of mRNA were analysed using the comparative Ct method. Each cDNA sample was amplified in doublet and all data are expressed as the mean ± SEM, *P ≤ 0.05, **P ≤ 0.01; (experimental vs the control animals)

The results of the RT-PCR performed on tissue samples collected from M-group animals revealed a variety of changes in the expression of particular gens between the control and experimental pigs in each stomach region studied (Fig. 7). In detail: The expression of mRNA encoding Tac 1 (substance P) was significantly increased only in the corpus tissues in the experimental animals (in relation to controls) (Fig. 7a), while in the remaining regions the increase was not statistically insignificant (Fig. 7b, c). The expression of NK1 receptor was significantly increased in the corpus samples in the experimental pigs (Fig. 7d). In the experimental animals, the expression of Nk2 was significantly decreased in the stomach antrum (in the area adjacent to the ulcer) (Fig. 7h), increased in the pylorus (Fig. 7i) and not significantly changed in the corpus (Fig. 7g). The increase in the expression of Nk3 receptor was statistically significant (in relation to the controls) in all the tissues studied in the experimental pigs (Fig. 7j–l).

## Discussion

The present study revealed that in animals with stomach antrum ulcerations the expression of substance P immunoreactivity increased in the myenteric neurons found in all gastric locations examined: in both, those directly adjacent to the ulcer (antrum) and in those placed proximal (in the corpus) and distal (in the pylorus) to the pathologically changed tissues. The same number of tissue slides (of the identical thickness) analyzed in the control and experimental animals and thus assessing comparable groups of PGP 9.5-immunoreactive perikarya as well as the results showing the increased level of SP in myenteric plexus neurons during intestinal inflammation (Swain et al. 1992) seem to exclude the possibility that the changes dealing with the number of SP-immunoreactive perikarya resulted from the loss of SP-negative neurons. Changes in the number of SP-immunoreactive submucosal perikarya were not statistically significant. The tracing experiments revealed the absence of SP-immunoreactivity in gastric descending neurons supplying the pyloric sphincter. The Real-Time PCR investigations disclosed different fluctuations in the expression of mRNA encoding Tac1 (substance P) and Nk1, Nk2, Nk3 receptors depending on the stomach region studied.

### Gastrointestinal tachykinins under physiological conditions

Although the family of tachykinins comprises several other peptides, as neurokinin A (including its N-terminally extended forms: neuropeptide K and neuropeptide M), neurokinin B and recently discovered hemokinin-1, the substance P is expressed most abundantly in the stomach tissues of various species (Ferri et al. 1989; Flatt et al. 1991; Hayashi et al. 1982; Holzer et al. 1980; Schmidt et al. 1991). The NKB is completely absent in the porcine stomach (Schmidt et al. 1996) while SP and NKA are present in stable and equal amounts (Flatt et al. 1991; Schmidt et al. 1991). The highly specific anti-substance P antibodies, already used to investigate porcine tissues, allowed for precise quantitative assessment of the number of SP-immunoreactive structures in the porcine stomach.

Tachykinins play a variety of regulatory functions in the gastrointestinal tract under physiological conditions. Their ability to influence the action of longitudinal and circular gastric muscular layers, and thereby the intragastric pressure, volume as well as gastric emptying has been comprehensively described (Schmidt and Holst 2000). Tachykinins also regulate the ion transport through epithelia (Lordal et al. 1996). They act via Nk1, Nk2, and Nk3 receptors, all of which have been found in the stomach, with the higher amounts of Nk2 (Tsuchida et al. 1990).

The present results obtained in the control pigs revealed substance P immunoreactivity in the myenteric and submucosal neurons as well as the expression of mRNA encoding SP, and Nk1, Nk2, Nk3 receptors in all the stomach locations studied. These data confirm the involvement of neuronal SP in the physiological regulation of all these parts of the porcine stomach. Interestingly, the percentage of the porcine SP-immunoreactive myenteric neurons increased towards the aboral direction, from about 12% in the corpus, through 15% in the antrum to reach 27% in the pylorus. Correspondingly, the extractable SP immunoreactivity in the human stomach increased towards the distal part (Ferri et al. 1989), and the highest concentrations of SP binding sites were found in the pyloric circular muscle of the feline stomach, as well (Rothstein et al. 1991). These data further confirm that SP is of major importance for the regulation of the activity of the pyloric sphincter in different mammals, including the pig.

### Substance P in gastrointestinal disorders

Another role played by SP-expressing enteric neurons is related to their morphological relationship with immune cells (Batbayar et al. 2003). Such intimate contacts provide bidirectional association between neural and immune functions (Elenkov 2008). Substance P is strongly involved in the regulation of inflammatory processes in different tissues (O’Connor et al. 2004), including the gastrointestinal tract (Mantyh et al. 1995, 1996). It can modulate gastritis via inducing neurogenic inflammation (Sipos et al. 2008; Larauche et al. 2004) and plays a key function in the immune cells activation, mainly via Nk1 receptor (Ho et al. 1997; Lai et al. 1998). The ability of SP to activate the transcription of pro-inflammatory genes in the immune cells (Bardelli et al. 2005; Koon et al. 2005) and initiate migration, adherence, lysosomal enzyme release, production and release of tumor necrosis factor alpha, cytokines and interleukins (IL-l, Il-6) (Bar-Shavit et al. 1980; Ho et al. 1996) indicates its essential role in the development and course of acute inflammation.

Moreover, the levels of SP and its receptors were significantly increased during chronic intestinal inflammation (Mantyh et al. 1988, 1989). The measurements of neuronal and non-neuronal SP levels in the stomach biopsies collected from patients with gastritis or duodenal ulcer revealed their fluctuations depending on the degree of the tissue injury (affected vs unaffected mucosa) and the type of pathological condition (gastritis vs ulcer) (Erin et al. 2012). In rodents, experimentally induced intestinal inflammation with application of parasites (*Trichinella spiralis*) resulted in an extensively increased excitability of jejunal myenteric neurons which was strictly correlated with the occurrence of disturbances in motility and secretion (Palmer et al. 1998). Other studies involving the same inflammatory model revealed the increased level of SP in myenteric neurons (Swain et al. 1992).

All these data strongly correlate with the increased number of myenteric SP-immunoreactive nerve cells observed in the present study. What is more, the present results unveiled the widespread reaction of the distanced myenteric perikarya in response to the stomach ulcer indicating sophisticated neuronal plasticity that employs SP as the modulatory peptide. Such extensive neuronal response may be at least partly due to the severe inflammatory reaction accompanying acute ulcer disease and the need to activate immune cells.

Gastric ulcerations localized in the distal part of the stomach are frequently accompanied by impaired gastric motility and problems with gastric emptying (Kanaizumi et al. 1989; Murray et al. 1967; Texter, Jr. et al. 1959). According to various scientists such disturbances may be related to the inflammatory stimulation of muscular layer activity (Kanaizumi et al. 1989) and/or impaired gastric innervation (Garret et al. 1966; Liebermann-Meffert and Allgower 1977, 1981). Recent studies have demonstrated alterations in the galaninergic innervation (Zalecki et al. 2016, 2018) and in the number/distribution of intramural descending neurons supplying the pyloric sphincter (Zalecki 2015) in acute antral ulcerations.

Investigations involving the application of botulinum toxin have identified a completely novel function of SP which has appeared to be a key factor for acetylcholine to initiate gastrointestinal smooth muscle contractions (Li et al. 2014). Moreover, botulinum toxin inhibited the release of SP from the enteric nerve terminals, thereby reducing contractile tension of the pyloric sphincter (Shao et al. 2015). Since the application of botulinum toxin is increasingly used in the treatment of spastic gastrointestinal smooth muscle disorders (Vittal and Pasricha 2006), the present results suggest the possible advantages of “botulin” therapy in patients with gastric emptying problems arising from pyloric canal ulcerations.

### Tac1, Nk1, Nk2, Nk3 mRNA expression changes in gastrointestinal disorders

The results of the Q-PCR revealed different changes in the expression of Tac1 (substance P) and Nk1, Nk2 and Nk3 receptors depending on the tissue localization. In the experimental pigs, a significant increase in mRNA encoding Tac1 was observed only in the stomach corpus, while in the remaining localizations, the increase was not significant. Although the derivation of tissue samples analyzed by immunofluorescence and Q-PCR was strictly correlated for all the animals studied, the mRNA homogenates contained different cell structures found in the stomach wall, including epithelia, muscles, and immune cells, while immunofluorescence and neuronal tracing investigations were strictly focused on the neuronal elements. Moreover, at the time points of the analysis, all the antral ulcers were fully developed, thus the first phase of inflammation and cell adaptation (with significant mRNA elevation) could have already been finished in some of the studied tissues. Both these aspects could explain a small inconsistency in the statistical significance of SP-related data acquired with immunofluorescence and Q-PCR analyses, respectively.

Changes in the expression of mRNA encoding Nk1, Nk2 and Nk3 receptors observed between examined sample localizations differed significantly between the experimental and control animals. The increase in mRNA expression in all the regions studied concerned only Nk3 receptor, while statistically significant increase in the expression of Nk1 receptor was observed only in the stomach corpus. Considering neuronal localization of both receptors within the gastrointestinal tract (Schmidt and Holst 2000) and their role in generating slow excitatory postsynaptic potentials in enteric neurons (Steinhoff et al. 2014; Johnson and Bornstein 2004) it can be speculated that sensitized neurons activate distinct signal transduction pathways in myenteric neuronal circuits what implies the increased muscular activity in the whole stomach of experimental animals. Although both receptors were also found to be involved in the regulation of inflammatory processes occurring in different peripheral tissues (Steinhoff et al. 2014), the only available data for the gastrointestinal tract deal with changes in the expression of Nk1 receptor (Renzi et al. 2000; Karagiannides et al. 2006; Bhatia et al. 1998). The only information on changes in the expression of Nk3 receptor in peripheral tissues is related to the female reproductive tract (Steinhoff et al. 2014). The present results contribute to this fragmentary knowledge and suggest the participation of Nk3 receptor in the extensive reaction of the stomach tissues to acute antral ulcerations. However, the functional significance of these relationships remains to be elucidated.

The present study has demonstrated significant quantitative variations in the expression of mRNA encoding Nk2 receptor between both groups of pigs, which were largely dependent on the stomach region studied. It can be speculated that such results are directly linked with the localization and function of Nk2 receptor in the gastrointestinal tract as well as with the unique role performed by the pylorus. Nk2 receptors are expressed on the smooth muscle cells (Schmidt and Holst 2000), and their activation leads to contractions of the gastrointestinal muscular layer. The pylorus, via its sphincter muscle, adjusts the gastric outflow to the physiological needs of the body. Thus, it is the key regulator of the stomach emptying process. The highest amounts of SP and Nk2 receptors determined in the human and cat (Ferri et al. 1989; Rothstein et al. 1991) pylorus under normal conditions correspond with the increased expression of Nk2 receptor mRNA found in the pyloric tissues observed in the present study. Furthermore, these results strictly correlate and additionally corroborate previously mentioned assumption on the involvement of tachykinins in gastric emptying problems observed in patients with antral ulcers.

The reduced expression of Nk2 receptor in the tissues adjacent to the ulcer seems to be a consequence of the muscular layer damage caused by the deeply penetrating ulcer. In the tissue samples from this region, a large number of smooth muscle cells was affected by the ulcer and some of them were atrophied or heavily damaged. Such conditions must have influenced the number of receptors expressed on these myocytes.

### Substance P in gastric descending neurons supplying the pyloric sphincter

The results obtained from the “tracing” group provided some interesting information on the lack of SP-immunoreactive intramural descending neurons supplying the pyloric sphincter in these pigs. The study involving the experimental denervation of the rat pylorus provided similar data (Lindestrom and Ekblad 2002). On the other hand, investigations performed in lambs have revealed the presence of as many as 61% of SP-immunoreactive intramural descending neurons innervating the pyloric sphincter (Mazzuoli et al. 2008). Since the present experiment and the study of Mazzuoli et al. (2008) were carried out with the same neuronal tracer (Fast Blue), the discrepancies observed probably result from interspecies differences. Moreover, about 43% of the total abomasal myenteric neuronal population in the lamb were SP-immunoreactive, while in the control pigs about 13–15% of stomach corpus and antrum myenteric neurons stained for this peptide. Lambs, like other ruminants, have a four-compartment stomach, which exhibits significant differences in morphology and function in relation to the one compartment stomach found in the majority of other mammals including humans, pigs and rats, and this could be the reason for the above-mentioned discrepancies.

## Summary

The present results seem to directly confirm the involvement of substance P and all its receptors in the enteric nerve regulation of the stomach function in physiological conditions as well as their contribution to the extensive reaction of stomach tissues to acute antral ulcerations. Increased number of SP-immunoreactive myenteric neurons in all studied parts of the porcine stomach, as a result of focused antral ulcers, clearly indicates the importance of such neurons for the neural plasticity. The inflammatory reaction accompanying the acute ulcer disease can contribute to triggering the widespread neuronal response. Interestingly, SP is not directly involved in the reaction of submucosal neurons and intrinsic gastric descending nerve pathways supplying pyloric sphincter in animals with antral ulcerations. The data obtained allow for the assumption that problems with the gastric motility and emptying observed in patients with gastric ulcerations are, at least partly, associated with the upregulation of substance P expression in the stomach myenteric neurons. Although this hypothesis requires further confirmation by pathophysiological studies, the present neuroanatomical data provide direction for further experiments. Considering the effective usage of tachykinin antagonists in medicine (as antiemetic drugs) (Quartara and Altamura 2006) and animal experiments showing significant clinical improvement in jejunitis after inhibition of SP synthesis/release (Agro and Stanisz 1993), further studies should be aimed at verifying whether application of SP-antagonists can be helpful in the treatment of the stomach ulcer disease.
